# Validación de un cuestionario sobre conocimientos y actitudes ante la mutilación genital femenina y su abordaje sanitario en estudiantes de enfermería: Estudio piloto

**DOI:** 10.23938/ASSN.1123

**Published:** 2025-06-30

**Authors:** María del Mar Pastor Bravo, Yusuly Mitkany Martínez Jurado, Souad Ayad Ouchen

**Affiliations:** 1 Universidad de Murcia Facultad de Enfermería El Palmar Murcia España; 2 Universidad Autónoma del Estado de México Facultad de Enfermería y Obstetricia Toluca de Lerdo México

**Keywords:** Mutilación Genital Femenina, Encuestas y Cuestionarios, Estudio de Validación, Conocimiento, Actitudes y Práctica en Salud, Estudiantes de Enfermería, Genital Mutilation Female, Surveys and Questionnaires, Validation Study, Health Knowledge Attitudes, Practice, Students Nursing

## Abstract

**Fundamento:**

Diseñar y validar un cuestionario autoadministrado que valore integralmente los conocimientos, actitudes y abordaje sanitario de la mutilación genital femenina en estudiantes de enfermería.

**Metodología:**

Estudio piloto transversal realizado con estudiantes de tercer curso del grado de Enfermería de la Facultad de Enfermería de la Universidad de Murcia, sede en Cartagena (España), mediante muestreo por conveniencia. El cuestionario se diseñó tras realizar una búsqueda bibliográfica de otros instrumentos que midieran conocimientos, actitudes y abordaje sanitario de la mutilación genital femenina. El comité de expertas valoró el contenido de forma individual y, mediante una discusión con las autoras, se determinaron las preguntas a incluir. La consistencia interna del cuestionario (uno de los parámetros de fiabilidad) se evaluó mediante el Alpha de Cronbach (α).

**Resultados:**

Se incluyeron 37 estudiantes, la mayoría mujeres (89,2%), con edades comprendidas entre los 20 y 56 años; el 27% no tenía experiencia profesional en el sector de la salud. Se obtuvo un cuestionario de 15 preguntas de respuesta única y múltiple divididas en tres dimensiones (Conocimientos, Actitudes, Abordaje sanitario de la mutilación genital femenina). La consistencia interna global del cuestionario fue buena (α= 0,838; IC95%: 31,00-35,59).

**Conclusiones:**

Los resultados preliminares sugieren que este cuestionario es un instrumento fiable y válido para valorar integralmente los conocimientos y actitudes frente a la mutilación genital femenina y su abordaje sanitario.

## Abstract (en)

**Background:**

This study aimed to develop and validate a self-administered questionnaire to comprehensively assess knowledge, attitudes, and healthcare approaches to female genital mutilation among nursing students.

**Methodology:**

A cross-sectional pilot study was conducted with third-year nursing students at the Faculty of Nursing, University of Murcia (Cartagena campus, Spain), using convenience sampling. The questionnaire was developed based on an extensive literature review of existing instruments assessing knowledge, attitudes, and healthcare practices related to female genital mutilation. An expert panel individually reviewed the content, and through consensus discussions with the authors, determined which items to include. Internal consistency was assessed using Cronbach’s alpha (α).

**Results:**

Thirty-seven students participated in the study, 89.2% of whom were females, with ages ranging from 20 to 56 years. About 27% reported no prior professional healthcare experience. The questionnaire comprised 15 single- and multiple-choice questions organized into three dimensions: Knowledge, Attitudes, and Health Approach to female genital mutilation. The overall internal consistency was good with a global α=0.838 (95% CI: 31.00-35.59).

**Conclusions:**

Preliminary findings suggest that this questionnaire is a valid and reliable tool for assessing nursing student’s knowledge of female genital mutilation, their attitudes towards it, and knowledge about its clinical approach.

## INTRODUCCIÓN

La Organización Mundial de la Salud (OMS) define la mutilación genital femenina (MGF) como aquella práctica que engloba todos los procedimientos de resección parcial o total de los genitales externos femeninos, así como otras lesiones realizadas sobre estos con fines no médicos^1^ constituyendo una violación del derecho a la salud y la integridad corporal^2^. Se realiza con mayor frecuencia en el período comprendido entre la infancia y la adolescencia^3^ y, actualmente más de 230 millones de mujeres y niñas han sufrido MGF en el mundo^4^. Según el censo de 2011, se estima que alrededor de 600.000 mujeres y niñas han sufrido la MGF en Europa^5^. En España existen 18.836 niñas y 80.279 mujeres procedentes de países donde se practica la MGF, como Senegal, Nigeria, Ghana y Mali, de las cuales 2.238 mujeres y 634 niñas residen en la Región de Murcia, una cifra que la sitúa en el noveno lugar entre las comunidades autónomas españolas con más población de este origen^6^.

La MGF puede tener numerosas consecuencias clasificándose en cuatro grandes grupos: 1) físicas: dolor intenso, hemorragia y shock a corto plazo, y quistes de inclusión, trastornos menstruales e infertilidad a largo plazo; 2) psicológicas: como trastorno del estrés postraumático^7^, depresión y ansiedad^8^; 3) sobre la función sexual: dispareunia, deficiente satisfacción sexual y ausencia de deseo sexual^7^ y 4) obstétricas: desgarros perineales, hemorragias posparto y parto prematuro^9^.

El personal sanitario desempeña un papel fundamental a la hora de prevenir, detectar y actuar ante posibles casos de MGF^10^ y especialmente el de atención primaria, ya que es el primer contacto con niñas o mujeres mutiladas genitalmente o en riesgo de serlo, debiendo estar debidamente formado y capacitado para el abordaje de dicha práctica^11^. Sin embargo, la falta de conocimientos sobre la MGF por parte de profesionales de salud y de estudiantes de enfermería sugiere que es un problema que puede pasar desapercibido y, por lo tanto, ser infradiagnosticado^12^. Por ello se hacen necesarios programas de formación que puedan evaluarse y sean efectivos, que partan de las necesidades del colectivo sanitario y se incluyan en el currículum del futuro personal sanitario.

A pesar de que existen escalas que evalúan conocimientos, actitudes y prácticas/percepciones de profesionales de la salud, algunas no fueron validadas^13^^-^^16^, mientras que otras son una adaptación de las ya existentes para aplicarlas en un país diferente al de validación^17^^,^^18^. Sin embargo, existe una escala validada en Estados Unidos para proveedores sanitarios que explora las características previamente mencionadas^19^. A nuestro saber, ninguna de las escalas previamente mencionadas evaluaron los conocimientos respecto al abordaje sanitario de la MGF ni se aplicaron a estudiantes de enfermería.

Por ello, el objetivo del estudio es diseñar y validar un cuestionario autoadministrado que valore integralmente los conocimientos y actitudes ante la MGF, así como el abordaje sanitario de esta, en estudiantes de enfermería.

## MATERIAL Y MÉTODOS

### Diseño y participantes

Estudio piloto transversal realizado en la Facultad de Enfermería de la Universidad de Murcia, sede en Cartagena, entre febrero y diciembre de 2024.

La población de estudio la constituían las 50 personas estudiantes de tercer curso del grado de Enfermería matriculadas en la asignatura *Enfermería y Salud de la Mujer* en la edición 2023/2024. Se incluyeron aquellos estudiantes que firmaron el consentimiento informado para poder acceder al cuestionario *online* mediante la herramienta *Encuestas* de la Universidad de Murcia.

El estudio cuenta con la autorización favorable número M10/2024/338 del Comité Ético de Investigación de la Universidad de Murcia.

### Proceso de elaboración y validación del cuestionario

Se desarrolló en cinco fases: 1) selección y redacción de preguntas, 2) análisis de la validez de contenido por expertos, 3) pilotaje cognitivo, 4) análisis de la respuesta al cuestionario, y 5) evaluación de la fiabilidad (consistencia interna).

### Selección y redacción de preguntas

Se llevó a cabo una búsqueda bibliográfica con el fin de identificar preguntas recogidas en otros instrumentos que midieran conocimientos y actitudes ante la mutilación genital femenina y su abordaje sanitario. Para ello se consultaron las siguientes bases de datos: SciELO, PubMed, Web of Science, CUIDEN y Cochrane Library. Para las bases de datos en español, se emplearon las palabras clave «Mutilación genital femenina», «Profesionales de la salud», «Actitudes» y «Práctica en Salud», y los DeCS: «Circuncisión femenina», «Conocimiento» y «Enfermería». Para las bases de datos en inglés, se emplearon las palabras clave «*Female genital mutilation*», «*Health professionals*», «*Nursing*» y «*Attitudes and Practice in Health*» y los MeSH: «*Circumcision*, *Female*», «*Knowledge*», «*Nursing*». Los límites establecidos fueron: texto completo, acceso abierto, idioma (español e inglés) y año de publicación (2019-2024). Además, la búsqueda se completó con la revisión de la bibliografía secundaria de los artículos seleccionados. Posteriormente se desarrolló una versión preliminar del cuestionario compuesto por 14 preguntas con opción de múltiple respuesta y con una única respuesta correcta.

### Análisis de la validez de contenido por expertos

Para la evaluación cualitativa de la validez de contenido realizada por expertos, se consultó a un grupo multidisciplinario de cuatro profesionales de la salud (tres enfermeras y una matrona) con una amplia experiencia en el tema de estudio. La función de las expertas consistió en evaluar individualmente las preguntas atendiendo a su relevancia y grado de comprensión, y evaluar de forma global el cuestionario y sus dimensiones. Las investigadoras mantuvieron una discusión a nivel individual con cada una de las expertas para determinar qué preguntas se incluirían finalmente en el cuestionario. Se introdujeron en el cuestionario todas las observaciones realizadas por las expertas.

### Pilotaje cognitivo

Se realizó una prueba piloto con las personas estudiantes de tercer curso del grado de Enfermería que aceptaron participar sobre la versión obtenida del instrumento tras la evaluación cualitativa por expertas. Se valoró la aceptabilidad del cuestionario, su comprensión y el tiempo empleado en su cumplimentación.

### Análisis de la respuesta al cuestionario

Se llevó a cabo un análisis de las respuestas del cuestionario con el objetivo de detectar aquellos con una frecuencia elevada de no respuesta.

### Evaluación de la fiabilidad (consistencia interna)

La consistencia interna, uno de los parámetros de la fiabilidad, se evaluó mediante el coeficiente Alpha de Cronbach (α), categorizándola de acuerdo con los criterios de George y Mallery: 0,90-0,95 excelente, >0,80 bueno, >0,70 aceptable, 0>0,60 cuestionable, y <0,50 inaceptable^20^. Las preguntas del estudio se describieron con moda, frecuencia y porcentaje. Las propiedades de medición del cuestionario se evaluaron mediante la lista de verificación de diseño de estudios de COSMIN^21^. Para el análisis de los datos se utilizó el programa IBM SPSS Statistics v.27.

## RESULTADOS

### Características sociodemográficas de la población

La muestra final de estudiantes que cumplimentaron el cuestionario fue de 37, un 89,2% mujeres, con edad media de 23,86 años y el 65% con experiencia nula o menor de un año en un sector relacionado con la salud; menos del 11% tenían más de 10 años de experiencia (Tabla 1).

**Tabla 1:** Características sociodemográficas de la muestra de estudiantes de tercer curso del Grado de Enfermería del curso académico 2023/24 (n=37)

Variables	n (%)
*Edad*	
Media	23,86 (rango: 26-56)
Moda	20
*Sexo*	
Femenino	33 (89,2)
Masculino	4 (10,8)
*Experiencia en un sector relacionado con la salud*	
Sin experiencia	10 (27,0)
< 1 año	14 (37,8)
1-10 años	9 (24,3)
10-20 años	1 (2,7)
20-30 años	3 (8,1)

### Selección y redacción de preguntas

Se obtuvieron 475 artículos de los cuales se seleccionaron siete^13^^-^^19^. De ellos, seis trataban sobre conocimientos sobre MGF, actitudes del personal sanitario ante la misma, el interés de dicha práctica y la necesidad de formación en ella, y uno trataba sobre los conocimientos y competencias culturales de la MGF^16^. Además, cuatro de los estudios presentaban instrumentos que han sido sometidos a validación^14^^,^^16^^,^^18^^,^^21^ aunque solo uno de ellos describió el proceso completo^19^ (Tabla 2).

**Tabla 2:** Características de los artículos seleccionados

Autoría	Proceso de validación	Preguntas /
País	Características psicométricas	Dimensiones
Idioma		Preguntas incluidas en nuestro cuestionario
Año		
Molina Gallego y col	- ND	- 10 preguntas
España	- ND	- 4 dimensiones:
Inglés		Conocimientos; Actitud; Interés; Formación
2021^13^		- 6
Correa Ventura y Báez Quintana	- Estadística descriptiva y análisis	- 10 preguntas
España	de contenido.	- 4 dimensiones:
Español	- ND	Conocimientos; Actitud; Interés; Formación
2021^14^		- 0
Mehriban y col	- ND	- 8 preguntas
Bangladesh, Países Bajos, Somalia, Malasia, Reino Unido	- ND	- 2 dimensiones:
Inglés		Conocimientos; Actitud
2023^15^		- 1
Mahgoub Ziyada y col	- Cita pilotaje. No aporta pruebas.	- 12 preguntas
Noruega	- ND	- 2 dimensiones:
Inglés		Conocimientos y competencias; Consultas
2023^16^		- 0
Kouta y col	- ND	- 25 preguntas
Chipre y Kenia	- ND	- 2 dimensiones:
Inglés		Conocimientos; Actitudes
2023^17^		- 1
Hamdy y col	- Cita pilotaje. No aporta pruebas.	- 38 preguntas
Egipto	- ND	- 4 dimensiones:
Inglés		Conocimiento; Actitudes; Práctica;
2023^18^		Razones para apoyarla
Marea y col	- Revisión de literatura, desarrollo de ítems de escala, revisión de expertos y pruebas previas.	- 75 preguntas
EEUU	- Análisis factorial exploratorio.	- 2 dimensiones:
Inglés		Conocimiento de las complicaciones; Actitudes de sanitarios
2021^19^		- 1

ND: No descrito.

**Tabla 3:** Proceso de selección y elaboración de preguntas

	Preguntas				
Dimensiones	De otros cuestionarios		Nuevos	Para validación	Incluidos en cuestionario
	Obtenidos	Seleccionados			
Conocimientos sobre MGF	52	7	1	8	8
Actitudes ante MGF	24	3	1	4	4
Conocimientos sobre abordaje sanitario de MGF	0	0	3	3	3

MGF: mutilación genital femenina.

Se obtuvieron 178 preguntas de la bibliografía relacionados con este estudio, seleccionando 10. Se consideró necesario incluir cuestiones relacionadas con la valoración de conocimientos sobre abordaje de la MGF mediante una serie de casos clínicos reales. En la Tabla 3 se detalla el proceso de selección y diseño de las preguntas.

### Análisis de validez de contenido por expertos

Tras la discusión con expertas, se propusieron diversas modificaciones en el cuestionario:

1. Definir cada uno de los tipos de MGF en lugar de numerar exclusivamente la tipología.
2. Añadir una pregunta relativa a la participación en la realización de dicha práctica para ahondar más en las actitudes.
3. Simplificar la redacción de aquellas preguntas relacionadas con los casos prácticos y agruparlas.

Finalmente, se obtuvo una versión del «Cuestionario sobre conocimientos, actitudes y abordaje sanitario de la mutilación genital femenina» (Anexo 1) compuesto por 15 preguntas, ocho de respuesta única, con tres opciones de respuesta posibles: sí, no, no sabe/no contesta (preguntas 1, 3, 5, 6, 7, 9, 10, 15), y siete preguntas de respuesta múltiple con cinco a once opciones de respuesta posibles (preguntas 2, 4, 8, 11, 12, 13, 14), constituyendo un total de 46 opciones de respuesta totales (Tabla 4).

**Tabla 4:** Número de opciones de respuesta por pregunta

Pregunta	Número de opciones de respuesta
1. ¿Conoce la Mutilación Genital Femenina (MGF)?	3
2. Si ha respondido SÍ a la pregunta anterior, ¿sabe cuántos tipos hay?:	4
3. ¿Realizando una valoración podría identificar una MGF?	3
4. ¿Cuáles piensa que son los motivos por los que se realiza? (Puede seleccionar múltiples respuestas):	4
5. ¿Cree que la práctica de la MGF debe respetarse como una opción por razones geográficas, culturales, religiosas u otras?	3
6. Si la MGF se hiciese con un control sanitario, ¿participaría como profesional?	3
7. ¿Ha detectado algún caso en el ejercicio de su profesión o conoce algún caso cercano?	3
8. ¿Qué problemas de salud se asocian con la MGF? (Puede seleccionar múltiples respuestas)	5
9. ¿Hay legislación respecto a la MGF en nuestro país?	3
10. ¿Conoce algún protocolo o guía de actuación?	3
11. ¿Cuál piensa que es la actuación recomendada ante una mujer con MGF? (Puede seleccionar múltiples respuestas)	4
12. ¿Qué actuación realizaría para una adecuada entrevista clínica ante una paciente originaria de un país donde se realiza la MGF? (Puede seleccionar múltiples respuestas)	7
13. ¿Cuál/es sería su actuación/es ante una niña menor de edad con factores de riesgo de MGF a la que se realiza una exploración genital y se comprueba que no está mutilada genitalmente? (Puede seleccionar múltiples respuestas):	7
14. ¿Cuál sería su actuación/es ante una mujer adulta embarazada que te cuenta que le realizaron la MGF en su infancia? (Puede seleccionar múltiples respuestas)	11
15. ¿Ha asistido a algún curso o charla sobre mutilación genital femenina (MGF)?	3

Dicho cuestionario incluye tres dimensiones:

- *Conocimientos sobre MGF*, compuesta por ocho preguntas (1, 2, 3, 4, 7, 8, 9, 10 y 15), que evalúa los conocimientos que tienen los estudiantes sobre la MGF.
- *Actitud ante la MGF*, compuesta por dos preguntas (5 y 6), que evalúa la postura de los estudiantes ante la práctica y su predisposición a participar en su realización.
- *Conocimientos con respecto al abordaje sanitario de la MGF*, compuesta por cuatro preguntas (11, 12, 13 y 14), que evalúa la actuación de los estudiantes ante diferentes casos prácticos de MGF.

Las preguntas que componen el cuestionario se han codificado de la siguiente manera: con valores comprendidos entre 0 y 2 (0=No, 1=Sí, 2=No sabe/No contesta) para las preguntas 1, 3, 5, 6, 7, 9, 10 y 15, y entre 0 y 1 (0=ausencia de respuesta, 1=opción marcada) para las preguntas 2, 4, 8, 11, 12, 13 y 14. Por lo tanto, la puntuación total del cuestionario es la suma de las puntuaciones obtenidas para cada pregunta oscilando entre 0 y 49 puntos correspondiendo 22 puntos a la dimensión Conocimiento, dos a la dimensión Actitud y 25 a la dimensión Abordaje sanitario de la MGF, asignándole un punto a cada respuesta correcta marcada. A mayor puntuación de la escala, mayor conocimiento.

### Pilotaje cognitivo

Tras el pilotaje cognitivo, no se detectó ninguna dificultad de comprensión por parte del estudiantado. Además, el tiempo para contestar el cuestionario osciló entre 2 y 7 minutos, siendo la media de 5,15 minutos.

### Análisis de la respuesta al cuestionario

La frecuencia de no respuesta al cuestionario fue nula, ya que los 37 participantes cumplimentaron todas las preguntas que componen el cuestionario. La pregunta número *10 ¿Conoce algún protocolo o guía de actuación?* presenta el mayor porcentaje de *No sabe/No contesta* (n=8; 21,6%).

### Análisis de la consistencia (fiabilidad) del cuestionario

Del análisis descriptivo de las 46 opciones posibles de respuesta se obtuvo un coeficiente Alpha de Cronbach global de 0,838 (IC95%: 31,00-35,59) (α>0,80); la dimensión con mayor consistencia interna fue *Abordaje sanitario de la MGF* (28 elementos; α=0,920; IC95%: 19,43-23,38) mientras que la menor consistencia se observó en las dimensiones *Conocimiento* (16 elementos; α=0,486; IC95%: 10,42-12,17) y *Actitud* (dos elementos; α=0,453; IC95%: 0,25-0,94). Las variables del estudio se describieron con frecuencia, porcentaje y moda (Tabla 5).

**Tabla 5:** Análisis descriptivo del cuestionario sobre sobre conocimientos y actitudes ante la mutilación genital femenina y su abordaje sanitario

Pregunta y opciones de respuesta		Moda	n (%)
1. ¿Conoce la Mutilación Genital Femenina (MGF)?		1	33 (89,2)
2. Si ha respondido SÍ a la pregunta anterior, ¿sabe cuántos tipos hay?:	2.1. Resección parcial o total del clítoris y/o prepucio	0	35 (94,6)
	2.2. Resección parcial o total del clítoris y los labios menores.	0	25 (67,6)
	2.3. Corte y recolocación de los labios menores o mayores, con o sin resección del clítoris.	0	33 (89,2)
	2.4. Todas las anteriores.	1	21 (56,8)
3. ¿Realizando una valoración podría identificar una MGF?		1	18 (48,6)
4. ¿Cuáles piensa que son los motivos por los que se realiza? (Puede seleccionar múltiples respuestas):	4.1. Religiosos	1	24 (64,9)
	4.2. Culturales	1	32 (86,5)
	4.3. Estéticos	0	25 (67,6)
	4.4. No sabe/No contesta	0	37 (100)
5. ¿Cree que la práctica de la MGF debe respetarse como una opción por razones geográficas, culturales, religiosas u otras?		0	33 (89,2)
6. Si la MGF se hiciese con un control sanitario, ¿participaría como profesional?		0	27 (73)
7. ¿Ha detectado algún caso en el ejercicio de su profesión o conoce algún caso cercano?		0	37 (100)
8. ¿Qué problemas de salud se asocian con la MGF? (Puede seleccionar múltiples respuestas):	8.1. Físicos	1	32 (86,5)
	8.2. Psíquicos	1	31 (81,3)
	8.3. Obstétricos	1	26 (70,3)
	8.4. Sexuales	1	37 (100)
	8.5. Sociales	1	28 (75,7)
9. ¿Hay legislación respecto a la MGF en nuestro país?		2	23 (62,2)
10. ¿Conoce algún protocolo o guía de actuación?		0	26 (70,3)
11. ¿Cuál piensa que es la actuación recomendada ante una mujer con MGF? (Puede seleccionar múltiples respuestas):	11.1. Educación	1	33 (89,2)
	11.2. Reportar a las autoridades	1	28 (75,7)
	11.3. Ignorar	0	37 (100)
	11.4. No sabe/No contesta	0	1 (2,7)
12. ¿Qué actuación realizaría para una adecuada entrevista clínica ante una paciente originaria de un país donde se realiza la MGF? (Puede seleccionar múltiples respuestas):	12.1. Registrar toda la información y actuaciones en la Historia Clínica.	1	34 (91,9)
	12.2. Informar y sensibilizar a la familia y a la menor en las consecuencias para la salud de la MGF.	1	34 (91,9)
	12.3. Identificar otras mujeres y niñas en riesgo en el entorno familiar.	1	35 (94,6)
	12.4. Comunicar el riesgo a la persona responsable de su seguimiento.	1	28 (75,7)
	12.5. Remitir hoja de notificación de maltrato infantil a Protección de Menores.	1	29 (78,4)
	12.6. Remitir Parte de lesiones al juzgado.	1	27 (73)
	12.7. Valorar si la familia tiene programado un viaje al país de origen.	1	26 (70,3)
13. ¿Cuál/es sería su actuación/es ante una niña menor de edad con factores de riesgo de MGF a la que se realiza una exploración genital y se comprueba que no está mutilada genitalmente? (Puede seleccionar múltiples respuestas):	13.1. Registrar toda la información y actuaciones en la Historia Clínica.	1	33 (89,2)
	13.2. Informar y sensibilizar a la familia y a la menor en las consecuencias para la salud de la MGF.	1	32 (86,5)
	13.3. Identificar otras mujeres y niñas en riesgo en el entorno familiar.	1	30 (81,1)
	13.4. Comunicar el riesgo a la persona responsable de su seguimiento.	1	32 (86,5)
	13.5. Remitir hoja de notificación de maltrato infantil a Protección de Menores.	0	23 (62.2)
	13.6. Remitir Parte de lesiones al juzgado.	0	26 (70,3)
	13.7.Valorar si la familia tiene programado un viaje al país de origen.	1	30 (81,1)
14. ¿Cuál sería su actuación/es ante una mujer adulta embarazada que te cuenta que le realizaron la MGF en su infancia? (Puede seleccionar múltiples respuestas)	14.1. Registrar toda la información y actuaciones en la Historia Clínica.	1	35 (94,6)
	14.2. Informar y sensibilizar a la mujer en las consecuencias para la salud de la MGF.	1	32 (86,5)
	14.3. Realizar la exploración genital y anamnesis antes de la semana 20 de gestación.	1	34 (91,9)
	14.4. Valorar el tipo de MGF y las consecuencias sobre la vía del parto.	1	35 (94,6)
	14.5. El parto será atendido por un equipo multidisciplinar del paritorio (matrona, ginecólogo, pediatra y anestesista) a diferencia de partos de mujeres sin MGF.	1	30 (81,1)
	14.6. En caso de necesitar desinfibulación, se realizará posteriormente a la semana 28 de gestación.	1	24 (64,9)
	14.7. Comunicar el riesgo a la persona responsable de su seguimiento.	1	25 (67,6)
	14.8. Registrar el tipo de MGF en la Cartilla maternal.	1	31 (83,8)
	14.9. Comunicar el riesgo de MGF en caso de que tenga una niña al responsable de su seguimiento.	1	30 (81,1)
	14.10. En el caso de tener una niña, indicar «niña en riesgo de mutilación genital femenina» en su historia clínica.	1	30 (81,1)
	14.11. Identificar otras mujeres y niñas en riesgo en el entorno familiar.	1	29 (78,4)
15. ¿Ha asistido a algún curso o charla sobre MGF?		0	31 (83,8)

## DISCUSIÓN

El cuestionario sobre conocimientos, actitudes y abordaje sanitario de la mutilación genital femenina diseñado en esta investigación muestra una consistencia interna buena (>0,80) en su puntuación global. Esta herramienta presenta mejoras con respecto al resto de cuestionarios localizados^13^^-^^19^. En primer lugar, valora integralmente la MGF en 3 dimensiones diferentes: conocimientos sobre MGF (tipología, identificación, causas, consecuencias, factores perpetuantes, legislación y protocolo de actuación), actitudes ante MGF y conocimientos sobre abordaje sanitario de la MGF (mediante casos prácticos). En segundo lugar, incluye una serie de casos clínicos reales que permiten detectar el grado de conocimiento de los participantes sobre el abordaje sanitario de dicha práctica. Por último, se trata de un instrumento validado para personal sanitario, concretamente para estudiantes de Enfermería, aunque en estudios posteriores se podría valorar su aplicación a otras categorías sanitarias.

Mediante la revisión de la literatura existente, se han encontrado pocos estudios e instrumentos actualizados que valoran aspectos relacionados con esta. En la literatura consultada, se han localizado siete cuestionarios con un objetivo similar al aquí propuesto, pero no fueron aplicados a estudiantes sanitarios ni evaluaron conocimientos respecto al abordaje sanitario de la MGF. Cuatro, de España, Somalia y Noruega, no estaban validados^13^^-^^16^, dos fueron una adaptación de cuestionarios existentes (Kaplan 2013, Cappon 2015 y Turkmani 2018)^17^^,^^18^ y uno fue validado en inglés (en Estados Unidos)^19^. En todos ellos se determina un nivel bajo de conocimientos con respecto a esta práctica destacando la necesidad de capacitar al personal sanitario para la detección y prevención de la MGF.

En España, destaca la obligatoriedad legal del personal sanitario de detectar y abordar los posibles casos de MGF para poner en conocimiento de la autoridad judicial la posible existencia de un acto delictivo^22^. Así mismo existe un Protocolo para la Prevención y Actuación Sanitaria ante la MGF en la Región de Murcia^23^ basado en el Protocolo Común para la actuación sanitaria ante la MGF. Dicho protocolo fue publicado en el Ministerio de Sanidad, Servicios Sociales e Igualdad en 2015^24^. Ante la obligatoriedad de detectar y abordar los casos de MGF, destaca la necesidad de crear nuevos instrumentos que valoren los conocimientos y actitudes ante la MGF, así como el abordaje sanitario de ésta en estudiantes de Enfermería y personal sanitario.

La MGF se trata de un tema sensible tanto en los países de origen de las mujeres como en Europa que genera importantes consecuencias físicas, psicológicas y sexuales en mujeres y niñas. El desconocimiento sobre dicha práctica y sus implicaciones legales en España se suma a las barreras culturales existentes en la atención sanitaria a las mujeres migrantes^25^. A pesar de que no todos los centros disponen de la figura del mediador cultural, cabe destacar el importante papel de las personas mediadoras interculturales. Puesto que proceden de la comunidad para la que están realizando la labor de mediación, ellas son las encargadas de realizar la interpretación de enlace para lograr una comprensión mutua y, además, explicar los respectivos códigos culturales que permiten comprender mejor las actitudes de las mujeres inmigrantes^26^. La información que este instrumento permite obtener resulta de vital importancia para conocer el grado de conocimiento de los estudiantes de enfermería y personal sanitario sobre la MGF, así como para plantear programas de formación sobre dicha práctica que doten de las herramientas necesarias para la prevención, detección y abordaje de la MGF.

Respecto a las fortalezas de este cuestionario, tras la realización de la investigación, se ha comprobado con la lista de verificación de diseño de estudios de COSMIN el cumplimiento de las siguientes características:

1. Proporciona un objetivo de investigación claro que incluye el constructo a medir, la población, el tipo de instrumento y las propiedades de medición de interés.
2. Validez de contenido por parte de profesionales utilizando un método cualitativo a nivel individual con cada experto para determinar qué preguntas se incluyen finalmente en el cuestionario.
3. La evaluación de la consistencia interna mediante el cálculo del Alpha de Cronbach.

Respecto a las limitaciones de este instrumento, en primer lugar, destaca el pequeño tamaño muestral (n=37) que hace que el estudio tenga baja potencia estadística y, en consecuencia, las estimaciones sean menos precisas. Además, el uso de la presente escala debe ser exclusivamente para estudiantes y personal sanitario para los cuales se ha validado ya que podría suponer una dificultad de comprensión en otro entorno. Asimismo, aunque se trata de un estudio de diseño y estudio de fiabilidad de una escala, solo se incluyen la valoración por un comité de expertos y los estudios de validez de contenido y de fiabilidad mediante la consistencia interna, por lo que no procedió realizar las siguientes propiedades de medición: validez estructural, validez transcultural/invariabilidad de la medición, fiabilidad, error de medición, validez de criterio, pruebas de hipótesis para la validez de constructo y capacidad de respuesta. Igualmente, a pesar de obtener un índice global bueno en la consistencia interna del cuestionario, dos de las tres dimensiones obtuvieron un alfa de Cronbach menor de 0,50, por lo que obtuvieron puntuaciones inferiores a las recomendadas para su aceptabilidad^19^. Se recomienda realizar futuros estudios con un mayor tamaño muestral, así como la realización del análisis factorial que permita identificar la totalidad de propiedades psicométricas del instrumento.

En conclusión, dicho cuestionario presenta buenas garantías respecto a la precisión con la que mide. Los resultados de análisis de fiabilidad han sido buenos en todas las preguntas que lo componen. Por lo que los resultados sugieren que el cuestionario sobre conocimientos, actitudes y abordaje sanitario de la mutilación genital femenina se trata de un instrumento fiable y válido que valora de forma integral los conocimientos sobre MGF, las actitudes ante esta y los conocimientos relacionado con su abordaje sanitario en estudiantes de Enfermería.

## Data Availability

Se encuentran disponibles bajo petición a la autora de correspondencia.

## ANEXOS

**Anexo I:** Cuestionario sobre conocimientos y actitudes ante la mutilación genital femenina y su abordaje sanitario

Cuestionario sobre conocimientos, actitudes y abordaje sanitario de la mutilación genital femenina
1. ¿Conoce la mutilación genital femenina (MGF)?
a. Sí
b. No
2. Si ha respondido SÍ a la pregunta anterior, ¿sabe cuántos tipos hay?
a. Resección parcial o total del clítoris y/o prepucio.
b. Resección parcial o total del clítoris y los labios menores.
c. Corte y recolocación de los labios menores o mayores, con o sin resección del clítoris.
d. Todas las anteriores.
3. ¿Realizando una valoración podría identificar una MGF?
a. Sí
b. No
c. No sabe/No contesta
4. ¿Cuáles piensa que son los motivos por los que se realiza? (Puede seleccionar múltiples respuestas)
a. Religiosos
b. Culturales
c. Estéticos
d. No sabe/No contesta
5. ¿Cree que la práctica de la MGF debe respetarse como una opción por razones geográficas, culturales, religiosas u otras?
a. Sí
b. No
c. No sabe/No contesta
6. Si la MGF se hiciese con un control sanitario, ¿participaría como profesional?
a. Sí
b. No
c. No sabe/No contesta
7. ¿Ha detectado algún caso en el ejercicio de su profesión o conoce algún caso cercano?
a. Sí
b. No
c. No sabe/No contesta
8. ¿Qué problemas de salud se asocian con la MGF? (Puede seleccionar múltiples respuestas)
a. Físicos
b. Psíquicos
c. Obstétricos
d. Sexuales
e. Sociales
9. ¿Hay legislación respecto a la MGF en nuestro país?
a. Sí
b. No
c. No sabe/No contesta
10. ¿Conoce algún protocolo o guía de actuación?
a. Sí
b. No
c. No sabe/No contesta
11. ¿Cuál piensa que es la actuación recomendada ante una mujer con MGF? (Puede seleccionar múltiples respuestas)
a. Educación
b. Reportar a las autoridades
c. Ignorar
d. No sabe/No contesta
12. ¿Qué actuación realizaría para una adecuada entrevista clínica ante una paciente originaria de un país donde se realiza la MGF? (Puede seleccionar múltiples respuestas)
a. Registrar toda la información y actuaciones en la Historia Clínica.
b. Informar y sensibilizar a la familia y a la menor en las consecuencias para la salud de la MGF.
c. Identificar otras mujeres y niñas en riesgo en el entorno familiar.
d. Comunicar el riesgo a la persona responsable de su seguimiento.
e. Remitir hoja de notificación de maltrato infantil a Protección de Menores.
f. Remitir Parte de lesiones al juzgado.
g. Valorar si la familia tiene programado un viaje al país de origen.
13. ¿Cuál/es sería su actuación/es ante una niña menor de edad con factores de riesgo de MGF a la que se realiza una exploración genital y se comprueba que no está mutilada genitalmente? (Puede seleccionar múltiples respuestas)
a. Registrar toda la información y actuaciones en la Historia Clínica.
b. Informar y sensibilizar a la familia y a la menor en las consecuencias para la salud de la MGF.
c. Identificar otras mujeres y niñas en riesgo en el entorno familiar.
d. Comunicar el riesgo a la persona responsable de su seguimiento.
e. Remitir hoja de notificación de maltrato infantil a Protección de Menores.
f. Remitir Parte de lesiones al juzgado.
g. Valorar si la familia tiene programado un viaje al país de origen.
14. ¿Cuál/es sería su actuación/es ante una mujer adulta embarazada que te cuenta que le realizaron la MGF en su infancia? (Puede seleccionar múltiples respuestas)
a. Registrar toda la información y actuaciones en la Historia Clínica.
b. Informar y sensibilizar a la mujer en las consecuencias para la salud de la MGF.
c. Realizar la exploración genital y anamnesis antes de la semana 20 de gestación.
d. Valorar el tipo de MGF y las consecuencias sobre la vía del parto.
e. El parto será atendido por un equipo multidisciplinar del paritorio (matrona, ginecólogo, pediatra y anestesista) a diferencia de partos de mujeres sin MGF.
f. En caso de necesitar desinfibulación, se realizará posteriormente a la semana 28 de gestación.
g. Comunicar el riesgo a la persona responsable de su seguimiento.
h. Registrar el tipo de MGF en la Cartilla maternal.
i. Comunicar el riesgo de MGF en caso de que tenga una niña al responsable de su seguimiento.
j. En el caso de tener una niña, indicar «niña en riesgo de mutilación genital femenina» en su historia clínica.
k. Identificar otras mujeres y niñas en riesgo en el entorno familiar.
15. ¿Ha asistido a algún curso o charla sobre mutilación genital femenina (MGF)?
a. Sí
b. No
